# Extracellular Vesicles Secreted by Hypoxic AC10 Cardiomyocytes Modulate Fibroblast Cell Motility

**DOI:** 10.3389/fcvm.2018.00152

**Published:** 2018-10-25

**Authors:** Imelda Ontoria-Oviedo, Akaitz Dorronsoro, Rafael Sánchez, Maria Ciria, Marta Gómez-Ferrer, Marc Buigues, Elena Grueso, Sandra Tejedor, Francisco García-García, Hernán González-King, Nahuel A. Garcia, Esteban Peiró-Molina, Pilar Sepúlveda

**Affiliations:** ^1^Regenerative Medicine and Heart Transplantation Unit, Instituto de Investigación Sanitaria La Fe, Valencia, Spain; ^2^Associated Unit for Cardiovascular Repair, Instituto de Investigación Sanitaria La Fe-Centro de Investigación Príncipe Felipe, Valencia, Spain; ^3^Bioinformatics and Biostatistics Unit, Centro de Investigación Principe Felipe, Valencia, Spain

**Keywords:** cardiomyocytes, fibroblasts, endothelial cells, extracellular vesicles, hypoxia, cellular communication

## Abstract

Extracellular vesicles (EVs) are small membrane vesicles secreted by most cell types with important roles in cell-to-cell communication. To assess their relevance in the context of heart ischemia, EVs isolated from the AC10 ventricular cardiomyocyte cell line (CM-EVs), exposed to normoxia (Nx) or hypoxia (Hx), were incubated with fibroblasts (Fb) and endothelial cells (EC). CM-EVs were studied using electron microscopy, nanoparticle tracking analysis (NTA), western blotting and proteomic analysis. Results showed that EVs had a strong preference to be internalized by EC over fibroblasts, suggesting an active exosome-based communication mechanism between CM and EC in the heart. In Matrigel tube-formation assays, Hx CM-EVs were inferior to Nx CM-EVs in angiogenesis. By contrast, in a wound-healing assay, wound closure was faster in fibroblasts treated with Hx CM-EVs than with Nx CM-EVs, supporting a pro-fibrotic effect of Hx CM-EVs. Overall, these observations were consistent with the different protein cargoes detected by proteomic analysis under Nx and Hx conditions and the biological pathways identified. The paracrine crosstalk between CM-EVs, Fb, and EC in different physiological conditions could account for the contribution of CM-EVs to cardiac remodeling after an ischemic insult.

## Introduction

Cardiac muscle is a highly adaptable tissue that responds to physiological challenges, in part, by releasing paracrine factors including extracellular vesicles (EVs) (1). Exosomes are small nanomembranous extracellular vesicles (30–150 nm in diameter) that contain a variety of molecules including cytokines, membrane trafficking molecules, chemokines, heat shock proteins, and also several types of RNA molecules. Due to their diverse cargo, exosomes play a central role in cell-to-cell communication and we and others have previously examined the role of cardiomyocyte-derived exosomes (CM-Exo) in different stress conditions, such as glucose starvation (2), inflammation (3, 4) or ethanol treatment (5). In response to ischemia, different cardiac cell types, including cardiomyocytes, release high amounts of exosomes and other types of EVs with different cargoes (6, 7). To characterize the changes in CM-EV cargo during ischemia, we performed a comparative proteomic analysis of EVs isolated from cardiomyocytes cultured in normoxia (Nx) or hypoxia (Hx), and mapped the biological processes activated in both conditions. We found that Hx increased the secretion of CM-EVs containing proteins related to wound healing. Consistent with this, we observed that Hx CM-EVs stimulated fibroblast motility. Our results shed more light on the contribution of CM-EVs to cardiac remodeling.

## Materials and methods

All procedures were approved by national and local ethical committees (reference number 2016/0192).

### Cell culture

The human ventricular cardiomyocyte cell line AC10 (8) was cultured in Dulbecco's Modified Eagle's Medium-F-12 (DMEM-F12, Gibco-Invitrogen®) supplemented with 10% fetal bovine serum (FBS, Gibco-Invitrogen®) and 1% penicillin/streptomycin (P/S, Millipore). Primary cultures of human umbilical cord vein endothelial cells (HUVEC) were obtained from Lonza and were grown in Endothelial Cell Growth Medium-2 (EGM-2) BulletKit™ (Lonza). Human coronary microvasculature (HCAEC; ATCC) endothelial cells were grown in Vascular Cell Basal Medium supplemented with the Endothelial Cell Growth Kit-VEGF (ATCC). Fibroblasts were obtained from skin biopsies after informed consent and were cultured in high-glucose DMEM (Thermo Fisher Scientific) supplemented with 10% FBS and 1% P/S. Cell were maintained under control conditions (Nx) in a humidified atmosphere at 37°C containing 5% CO_2_. AC10 cells cultured in Hx were incubated at 2% O_2_ for 48 h with glucose deprivation during the first 4 h and DMEM/F12 with depleted FBS during the next 44 h at 2% O_2_. Depleted FBS was generated by ultracentrifugation of an FBS and DMEM/F12 (1:1) mix at 100,000 *g* for 16 h. The induction of Hx in cell cultures was monitored by stabilization of hypoxia inducible factor-1 alpha (HIF-1α) expression and cell viability reduction (Figure S1).

### Extracellular vesicle purification

We used ultracentrifugation without sucrose-gradient centrifugation step to isolate EVs from cardiomyocytes. Accordingly, we refer to the isolated pool of vesicles as CM-EVs and not CM-exosomes. Approximately 150 mL of culture media was collected and EVs were isolated by several ultracentrifugation steps as described (9). Briefly, supernatants were centrifuged first at 2,000 *g* for 20 min (Eppendorf 5804 benchtop centrifuge, A-4-62 rotor), 10,000 *g* for 70 min (Hitachi CP100NX centrifuge, Beckman Coulter 50.2 Ti rotor) and subsequently filtered manually through a 0.22 μm filter to eliminate cell debris using a syringe. Then, EVs were pelleted by ultracentrifugation at 110,000 *g* for 120 min (Hitachi CP100NX centrifuge, Beckman Coulter 50.2 Ti rotor), filtered through a 0.22 μm filter to maintain sterility and ultracentrifuged again at 110,000 *g* for 120 min (Hitachi CP100NX centrifuge, Beckman Coulter 50.2 Ti rotor). The manipulation of EV extracts was performed in a laminar flow hood to preserve sterility. EV protein concentration was determined with the Pierce BCA Protein Assay Kit (ThermoFisher Scientific) to ensure equal amounts of protein samples. EVs were suspended in RIPA buffer [1% NP40, 0.5% deoxycholate, 0.1% sodium dodecyl sulfate in Tris-buffered saline (TBS), Sigma-Aldrich] for western blotting or phosphate buffered saline (PBS) for nanoparticle tracking, electron microscopy, flow cytometry, proteomic and functional analysis.

### Extracellualar vesicle incorporation experiments

Capture of labeled EVs by fibroblasts and EC was performed using procedures modified from previous reports (10). EVs were fluorescently stained with carboxyfluorescein diacetate succinimidyl diester (CFSE; 5 μM) (ThermoFisher Scientific) for 15 min at 37°C, and unincorporated dye was removed *via* ultracentrifugation. As a negative control to normalize, the same amount of PBS was added to CFSE to monitor unincorporated dye carried over after the staining steps (PBS control). Cells were seeded at 50% confluency in 24-well plates and incubated with CFSE-labeled EVs at 2 μg/mL (5.00 E + 08 particles/mL). After 3 h of incubation, cells were washed twice in cold PBS, trypsinized and analyzed by flow cytometry using a FACS Canto II at the Cytomics Unit of the Instituto de Investigación Sanitaria, La Fe.

### Western blot analysis

EVs or cells were lysed in 100 μL of RIPA buffer containing protease (Complete, Sigma-Aldrich) and phosphatase (PhosSTOP, Sigma-Aldrich) inhibitors. Equal amounts of protein samples were suspended in non-reducing Laemmli sample buffer (BioRad) and denatured at 100°C for 5 min. Proteins were separated on 10% SDS-polyacrylamide gels and transferred to polyvinylidene difluoride membranes (Immobilon-P; Millipore). Membranes were blocked with TBS containing 5% (w/v) nonfat dry milk powder with 0.1% Tween-20. Antibodies used were anti-CD9 (Ab 92726, Abcam), anti-TGS 101 (Santa Cruz. Sc-7964), anti-Alix (Santa Cruz. Sc-53538) and HIF-1α (610958, BD biosciences). Detection was carried out using peroxidase-conjugated secondary antibodies with the ECL Plus Reagent (Amersham, GE Healthcare, Munich, Germany). Proteins were visualized using an Amershan Imager 600 (GE Healthcare) and quantified with ImageJ software (NIH).

### Nanoparticle tracking analysis

EV size distribution and quantification of vesicles was analyzed by Nanoparticle tracking analysis (NTA) using a NanoSight NS3000 System (Malvern Instruments, UK) Samples were suspended in 0.22 μm pre-filtered PBS and dilutions between 1:1,000 and 1:10,000 were used to achieve a particle count between 2 × 10^8^ and 2 × 10^9^ per mL. Measurement of the diameter was performed on 3 independent experiments and showed as mode ± standard deviation.

### Electron microscopy

Electron microscopy was performed as described previously (11). Briefly, isolated EVs were diluted in PBS, loaded onto Formwar carbon-coated grids, contrasted with 2% uranyl acetate and finally examined with a FEI Tecnai G2 Spirit transmission electron microscope. Images were acquired using a Morada CCD Camera (Olympus Soft Image Solutions GmbH). The size of the EVs was quantified using ImageJ. The diameter of a minimum of 25 EVs in different fields was measured in 3 independent experiments and the median ± standard deviation showed.

### Cell proliferation

To test whether purified EVs from AC10 cells grown under Nx and Hx conditions altered cell viability, AC10 cells, fibroblasts and HUVEC were cultured at a density of 5 × 10^3^ per well of a 96-well plate. Cells were incubated for 48 h with 30 μg/mL (7.00 E + 09 particles/mL) of purified EVs and the Cell Counting Kit-8 (CCK-8) assay was used to measure proliferation following the manufacturer's instructions. The optical density of the cultures was measured at 450 nm in each well 4 h after incubation with the CCK-8 assay solution.

### Tube formation assay

Capillary-like tube formation, as a readout of angiogenesis, was measured using the tube formation assay as previously described (12). In total, 1.2 × 10^4^ HUVEC were seeded per well into 96-well plates precoated with 50 μl of growth factor-reduced Matrigel (BD Biosciences). Cells were incubated for 6 h with 30 μg/mL (7.00 E + 09 particles/mL) of purified EVs from Nx and Hx conditions to evaluate formation of tube-like structures. The day after, images from three different viewing fields per sample were taken using an inverted microscope (Leica DM6000) with a 10 × magnification. Images were analyzed with WimTube online software (WimTube: Tube Formation Assay Image Analysis Solution. Release 4.0. https://www.wimasis.com/en/WimTube).

### *In vitro* scratch assay

The scratch assay is a good and valid method to measure cell migration *in vitro* (13). Human fibroblasts were seeded in 12-well culture plates at a density of 1.2 × 10^5^ cells/well. The day after, a straight line was created on the monolayer with a pipette tip. Cells were washed once with PBS to remove the debris and treatments were added in 1 mL of corresponding medium. In total, 30 μg/mL (7.00 E + 09 particles/mL) of EVs obtained from AC10 cells cultured in Nx and Hx conditions were added to the cells. The capacity of the cells to migrate and invade the denuded area was tracked using time-lapse microscopy during 24 h. Image analysis was performed using ImageJ software.

### Proteomic analysis

Proteomic analysis was carried out as described previously (11). Briefly, CM-EVs were harvested from 75 mL of culture medium, suspended in RIPA buffer, and protein concentration was determined using the Qubit® Protein Assay Kit (Invitrogen). A total of 30 μg of protein extract in Laemmli buffer was subjected to 12.5% acrylamide 1D SDS-PAGE electrophoresis and proteins were digested with trypsin. Digestion was stopped with 1% trifluoroacetic acid and 5 μL of each sample was loaded for liquid chromatography and tandem mass spectrometry (LC-MS/MS). Data were analyzed following the ProteinPilot default parameters (ProteinPilot v4.5. search engine, ABSciex). Proteins were arranged by the Unused Protein Score, which is a measure of the protein confidence calculated from the peptide confidence for peptides from spectra.

Functional enrichment analysis (14, 15) was performed to detect associations of Gene Ontology (GO) terms (16) for each experimental group. This functional characterization method allowed us to determine whether a set of functions was over-represented in a subset of proteins. Significant biological processes were summarized and represented by TreeMaps using the REVIGO webtool (17), which enabled the comparison of results between groups and is especially useful in revealing functional patterns.

### Statistical analysis

Data are represented as mean ± standard error (SE) unless otherwise is specified. Comparisons between experimental conditions were performed with Student's unpaired *t*-test. Analyses were conducted with GraphPad Prism 5 software (GraphPad Software Inc., La Jolla, CA). Differences were considered statistically significant at *p* < 0.05 with a 95% confidence interval.

## Results

**Figure 1:**
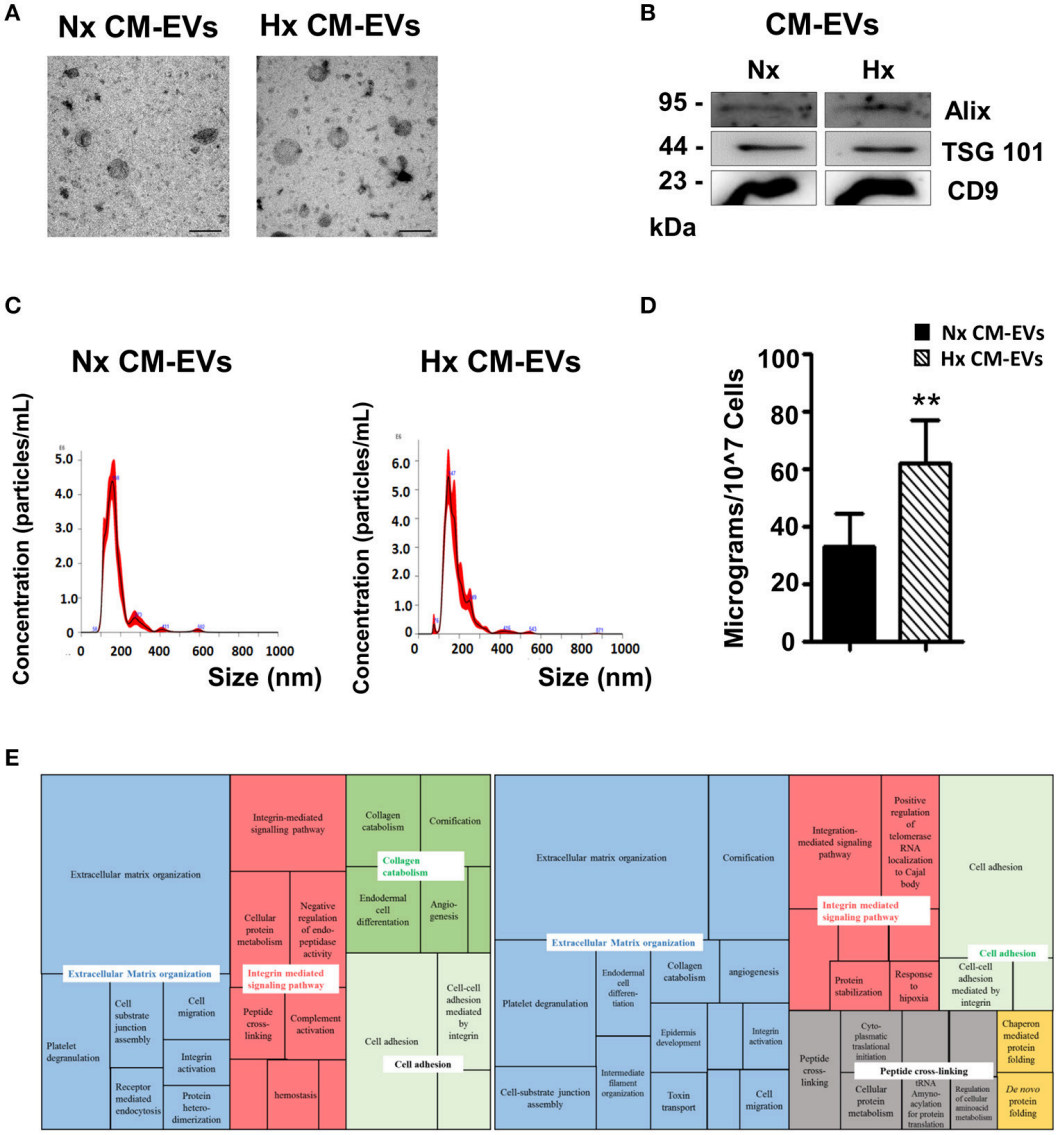
Characterization of cardiomyocyte-derived extracellular vesicles. **(A)** Representative electron microscopy images of isolated extracellular vesicles (EVs) collected from cardiomyocyte (CM) cultures in normoxia (Nx) or hypoxia (Hx) (*n* = 3). Scale bars = 200 nm. **(B)** Representative western blot of common proteins found in EVs. EVs were lysed in RIPA buffer with complete protease inhibitors. Proteins were separated on 10% SDS-polyacrylamide gels and transferred to polyvinylidene difluoride membranes. **(C)** Representative images of EVs analyzed on the NanoSight NS300 instrument: particles/mL on vertical axis and size in nanometers (nm) on horizontal axis (*n* = 5). **(D)** Protein quantification of EVs harvested from equal amounts of conditioned medium (*n* = 3, **p < 0.01). **(E)** Treemap diagram of biological processes in Nx and Hx CM-EVs using REVIGO after proteomic analysis. Extracellular matrix organization (blue), integrin-mediated signaling pathway (red), collagen catabolism (dark green), cell adhesion (light green), peptide cross-linking (gray), and protein folding (yellow).

Analysis of CM-EVs by western blotting for the assessment of the common exosomal markers CD9, TSG101, and ALIX (Figure 1B), showed no difference between EVs isolated from Nx or Hx conditions. In addition morphological analysis (Figure 1A) and size measurement by electron microscopy showed no significant neither difference (Nx: 102.9 ± 19.81 μm vs. Hx: 88.49 ± 24.35 μm). Further characterization using the NanoSight instrument revealed that the majority of EVs under both conditions were ~150 nm in size (Nx: 147.9 ± 14 μm vs. Hx: 136.75 ± 13.78 μm; Figure 1C). Total protein analysis of the EV extracts showed that Hx significantly increased the secretion of CM-EVs (Figure 1D), as previously described (7). To functionally characterize the protein content of CM-EVs after Nx or Hx, we used LC-MS/MS. Protein contaminants from the cell culture medium (serum albumin and keratin) were removed from the analysis. Although an equivalent amount of total protein was used (30 μg), a broader range of proteins was observed in EVs recovered from Hx cultures (Tables S1, S2): 55 proteins were identified in Nx and 99 in Hx conditions. Among the identified proteins, we found abundant extracellular matrix (ECM) proteins including collagens and laminins, and also integrins, tetraspanins, ribosomal proteins and cardiac-specific proteins. Of note, multiple relevant proteins were found only under Hx conditions, including cardiogenic and cardioprotective proteins (Dickkopf-related protein 1, neuropilin 1 and netrin 4) and proteins related to cellular stress (ATP-citrate synthase, fatty acid synthase, X-ray repair cross-complementary protein 5 and aminopeptidase N). As described previously, Hx can induce the release of cardiac EVs that contain proteins with opposing activities (6). We further mapped the biological processes and metabolic pathways represented by CM-EVs proteins in Nx and Hx, finding 101 biological processes enriched in Nx and 124 in Hx (*P* < 0.05; Tables S3, S4). The most significant biological processes identified in both conditions were visualized in a Treemap diagram using REVIGO (Figure 1E). CM-EVs secreted in Nx conditions contained protein cargo involved in ECM organization, integrin-signaling, collagen catabolism, and cell adhesion and cell migration, whereas additional biological processes were over-represented in Hx CM-EVs, such as protein folding and peptide cross-linking, including pathways involved in chaperone activity and protein synthesis (Figure 1E).

**Figure 2:**
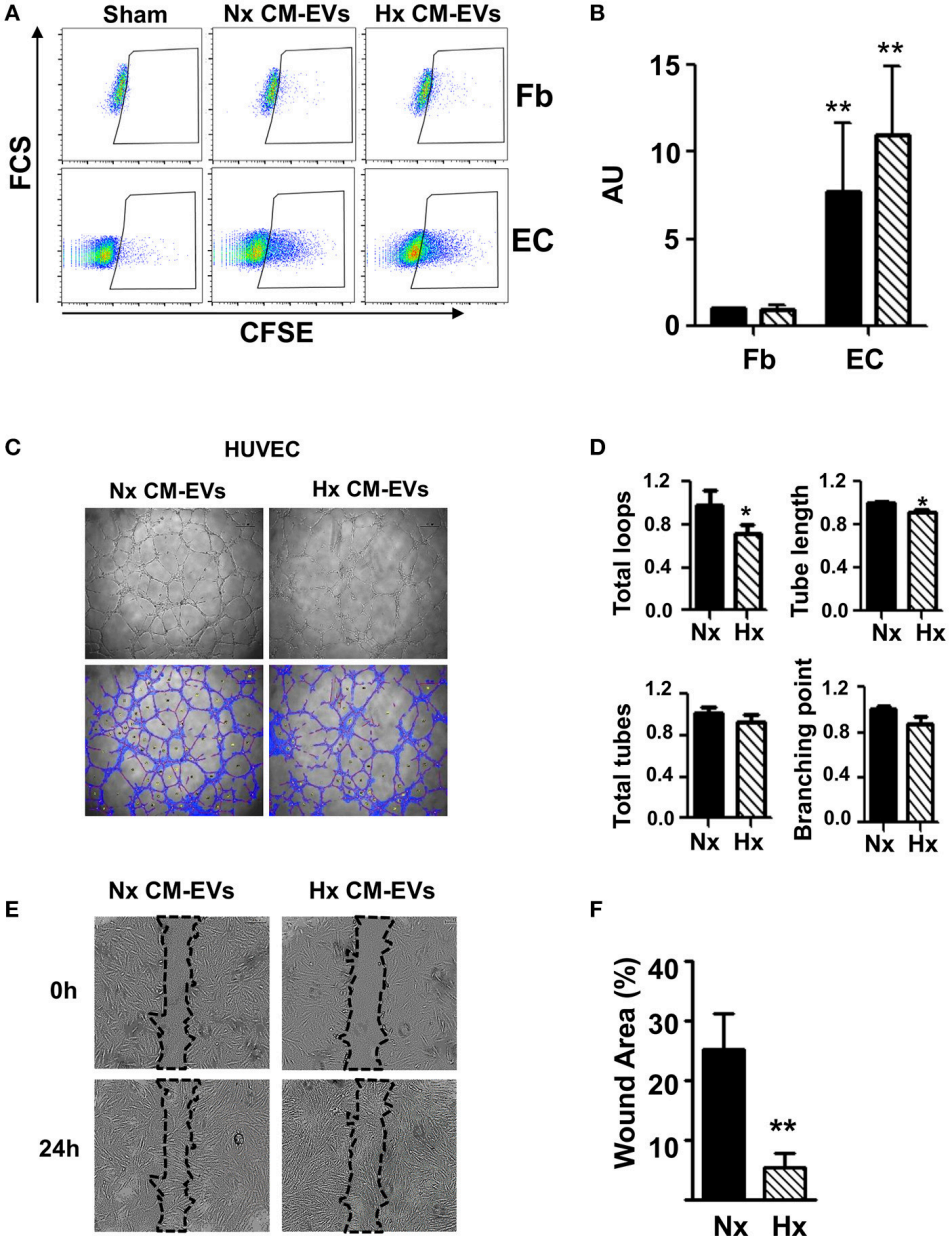
Functional analysis of cardiomyocyte-derived extracellular vesicles. **(A)** Representative images of flow cytometry analysis of non-conditioned media derived EVs stained (Sham) with CFSE, normoxia (Nx) and hypoxia (Hx) cardiomyocyte-derived extracellular vesicle (CM-EV) captured by fibroblasts (Fb) and endothelial cells (EC). **(B)** Flow cytometry quantification of fluorescence resulting from the incorporation of CFSE-labeled CM-EVs in Nx (black bars) and Hx (dashed bars) (*n* = 4, **P < 0.01). Data normalized to control condition. **(C)** Representative images of tube formation after 6 h of culture in the presence of Nx and Hx CM-EVs. **(D)** Quantification of total loops, loop length, total tubes and number of branching points from images taken after 6 h culture. Results are expressed in arbitrary units (*n* = 3, *P < 0.05). **(E)** Representative images of scratch assay after 24 h of culture in the presence of Nx and Hx CM-EVs. **(F)** Quantification of wound closure from images taken after 24 h culture normalized to initial wound area (*n* = 6, **P < 0.01).

To evaluate the functional activity of CM-EVs, we used CFSE to stain equal amounts of CM-EVs from both conditions and added them to fibroblast or HUVEC cultures to explore their ability to capture vesicles. Fluorescent cells resulting from the internalization of EVs were then quantified by flow cytometry. Results showed that CM-EVs had a stronger preference to be internalized in HUVEC than in fibroblasts, which was independent of culture conditions (Figures 2A,B), suggesting an active exosome-based communication mechanism between these cell types in the heart. To further explore this phenomenon, we added CM-EVs from Nx and Hx conditions to EC and evaluated their functional impact in a tube formation assay. We found that tube length and total number of loops in EC was lower when CM-EVs from Hx conditions were used than when Nx CM-EVs were used (Figures 2C,D), although no significant differences were found in the other parameters analyzed, such as total number of tubes and number of branching points in vascular nets (Figures 2C,D). Similar results were obtained when CM-EVs were added to HCAEC, in which tube length, number of total tubes and also branching points were significantly lower when Hx CM-Evs were used (Figure S2). To analyze the intercellular cross-talk between cardiomyocytes and fibroblasts mediated by CM-EVs, we evaluated the ability of these vesicles to modify the motility of fibroblasts in a wound healing assay. We observed accelerated wound closure when CM-EVs isolated under Hx conditions were added to fibroblasts as compared with Nx CM-EVs counterparts (Figures 2E,F). This occurred in the absence of any pro-mitotic effects of the CM-EVs (Figure S3). These responses of fibroblasts and EC to Hx CM-EVs might account for the cellular events observed during post-ischemic remodeling of the heart.

## Discussion

Cardiac cells, including cardiomyocytes, fibroblasts and EC, act in a coordinated fashion to support cardiac function; hence, an efficient intra-cardiac communication system is needed to control heart integrity in health and disease (6). Similar to many other eukaryotic cells, cardiac cells can release EVs, and changes in the amount and composition of these vesicles have been related to changes in physiological or pathological conditions (6, 18). The interplay between different cell populations mediated by EVs, including cardiac resident progenitor cells or cardiac progenitors, in homeostasis and pathological conditions is currently the object of intense investigation (19–21). A recent study exploring the functional activity of EVs isolated from iPSC-derived cardiovascular progenitors showed that these vesicles improved the survival of cardiac myoblasts and promoted tube formation in HUVEC *in vitro*, and also enhanced cardiac function *in vivo* in a murine model of chronic heart failure (22). In the present study, we sought to assess these biological processes by functionally analyzing EVs secreted by an adult cardiomyocyte-derived cell line (AC10) cultured in Nx and Hx conditions. We found that both Nx- and Hx-derived CM-EVs were loaded with ECM components including different types of collagens and laminins, which are typically secreted by interstitial cells to modulate normal cardiac growth and remodeling. Hx-derived CM-EVs also contained proteins related to chaperone activity and aminoacylation of proteins including T-complex protein 1 subunits α and γ and the aminoacyl-tRNA synthetase multienzyme complex. Another protein detected in Hx-derived CM-EVs was the tissue factor pathway inhibitor TFPI1, which has been shown to correlate with circulating fibrinogen levels and coronary artery disease (23), suggesting that CM-EVs might contribute to blood coagulation under similar *in vivo* conditions. GO analysis revealed the enrichment of terms associated with ECM organization, integrin-mediated signaling pathways and cell adhesion common to both Nx and Hx culture. Also, we found a major association of Hx-derived CM-EVs with two biological processes, peptide cross-linking and chaperone-mediated protein folding, which have been described in other cell types in response to pro-apoptotic stress (24). Nevertheless, our proteomic assay failed to find significant amounts of apoptotic proteins or biological processes related to apoptotic cascades, suggesting residual presence of apoptosis related vesicles in our preparations. However, since the presence of the mentioned vesicles has not been discarded, it is possible that observed effects on fibroblast and endothelial cells are partially trigger by apoptosis related vesicles. Further characterization of the secreted EVs will clarify the signaling processes involve in the effect of these EVs on the cardiac cells.

Low levels of oxygen following myocardial infarction account for the massive destruction of cardiomyocytes and EC in the myocardium. We show here a preferential EV-mediated communication between cardiomyocytes and EC, both in Nx and Hx conditions. Using a tube-forming assay, we observed lower tube formation in EC treated with Hx CM-EVs when compared with Nx CM-EVs. This result suggests that CM-EVs play a role in angiogenesis. Proteomic analysis revealed that both Nx- and Hx-derived EVs carry pro-angiogenic proteins, such as TGF-β and VEGF-C, but GO analysis indicated that Hx-derived EVs are less enriched in these factors than Nx EVs. This correlates with the results of the tube formation assay and could be related to the stress conditions of the parental cells. In this regard, the addition of CM-EVs from diabetic rats to EC cultures inhibited proliferation, migration and tube-like formation (25). Nevertheless, it has recently been described that rat CM-EVs obtained under Hx promote angiogenesis (26). The differences in regenerative potential between human and murine cells could account for these discrepancies, and further experiments are needed to elucidate these processes in detail.

To analyze the functional activity of CM-EVs on fibroblasts, we used a scratch assay to measure cell migration, finding that CM-EVs isolated from Hx cultures promoted fibroblast motility. This accelerated wound closure is consistent with the pro-fibrotic reaction in the heart after an ischemic insult. Similar deleterious effects exerted by CM through paracrine mechanisms have been described in other pathological conditions. For example, hypertrophic remodeling and fibrosis occurs in mice subjected to aortic constriction, which could be attenuated by cardiac-specific over-expression of the β3-adrenergic receptor (β3AR). The analysis of the secretome of β3AR-stimulated cardiac myocytes identified connective tissue growth factor as the main pro-fibrotic paracrine factor, which was reduced under β3AR stimulation (27). In another study, it was reported that diabetic (db/db) mice subjected to acute exercise increased their number of EVs in the heart containing miRNAs that reduce the content of MMP9, a metalloproteinase involved in cardiac fibrosis, with the consequent mitigation of this process (28). Similarly, in our proteomic analysis, we found disintegrin, metalloproteinase domain-containing protein 9 (ADAM9), laminin subunit gamma-1, TGFβI, fibronectin, laminin subunit alpha-5 and several subtypes of collagen-alpha protein in CM-EVs from Hx conditions; all of which are related to cell adhesion, cell-matrix interactions and cell motility, and that could account for the pro-fibrotic effects of CM-EVs. Overall, the results presented here contribute to the emerging picture of how CM-EVs function in cardiac remodeling after an ischemic insult. The differences between human and animal model systems should, however, be taken in consideration for the functional analysis of these vesicles.

## Limitations of the study

The major limitation of this study is the use of the AC10 cell line rather than primary cardiomyocyte cultures. However, the enormous number of cells needed for the isolation of EVs to perform the experiments would not have been feasible using primary cardiomyocytes and thus we decided to use AC10 cells. Another limitation of the study is that we did not verify the presence of proteins detected in the proteomic analysis by immunogold electron microscopy or alternative techniques, and so we cannot be sure that the proteins detected by LC-MS/MS are internalized in EVs and not non-specifically attached to isolated EVs. Finally, the low but unavoidable contamination of EV extracts with EVs originating from FBS used in cell culture should be considered. However, since we compared EVs derived from cell cultures in Nx and Hx conditions using the same batch of FBS, the differences observed in this study cannot be due to contamination with EVs from serum.

## Author contributions

IO-O and AD: conception and design of the study, acquisition of data, and analysis and interpretation of data; RS, MC, MG-F, MB, EG, ST, HG-K, NG, and FG-G: acquisition and analysis of data; IO-O, AD, NG, and EP-M: critical revision of the manuscript; PS: manuscript drafting, critical revision, and final approval of the version to be submitted.

### Conflict of interest statement

The authors declare that the research was conducted in the absence of any commercial or financial relationships that could be construed as a potential conflict of interest.

## Supplementary material

Figure S1 **(A)** Representative western blot images of proteins extracted from AC10 cell cultures in normoxia or hypoxia (2% O_2_). Lanes were loaded with equal amounts of protein and incubated with a hypoxia inducible factor-1 antibody. Signals were quantified by densitometry (*n* = 3). **(B)** Quantification of viable AC10 by CCK8 after different culture conditions used to isolate EVs.

Figure S2 Tube formation assay of human coronary artery endothelial cells (HCAEC). **(A)** Representative images of tube formation after 6 h of culture in the presence of normoxia (Nx) or hypoxia (Hx) cardiomyocyte-derived extracellular vesicles. **(B)** Quantification of total loops, tube length, total tubes and number of branching points from images taken after 6 h culture. Results are expressed in arbitrary units (*n* = 3, ^*^*P* < 0.05).

Figure S3 Cell proliferation assay of AC10, fibroblasts and human umbilical cord vein cells (HUVEC). Representative example of cell proliferation measured by CCK-8 assay after 48 h of culture in presence of Nx and Hx CM-EVs. Results are presented in arbitrary units (*n* = 3).

Table S1 Protein identification in cardiomyocyte-derived extracellular vesicles in normoxia.

Table S2 Protein identification in cardiomyocyte-derived extracellular vesicles in hypoxia.

Table S3 Gene ontology biological processes for proteins identified in extracellular vesicles derived in normoxia.

Table S4 Gene ontology biological processes for proteins identified in extracellular vesicles derived in hypoxia.

## References

[1] Safdar A Saleem A Tarnopolsky MA. The potential of endurance exercise-derived exosomes to treat metabolic diseases. Nat Rev Endocrinol. (2016) 12:504–17. 10.1038/nrendo.2016.76 27230949

[2] Garcia NA Moncayo-Arlandi J Sepulveda P Diez-Juan A. Cardiomyocyte exosomes regulate glycolytic flux in endothelium by direct transfer of GLUT transporters and glycolytic enzymes. Cardiovasc Res. (2016) 109:397–408. 10.1093/cvr/cvv260 26609058

[3] Gennebäck N Hellman U Malm L Larsson G Ronquist G Waldenström A. Growth factor stimulation of cardiomyocytes induces changes in the transcriptional contents of secreted exosomes. J Extracell Vesicles (2013) 2:20167. 10.3402/jev.v2i0.20167 24009898 PMC3760655

[4] Hirsch E Hilfiker-Kleiner D Balligand JL Tarone G De Windt L Bauersachs J. Interaction of the heart and its close and distant neighbours: report of the Meeting of the ESC Working Groups Myocardial Function and Cellular Biology. Cardiovasc Res. (2013) 99:595–9. 10.1093/cvr/cvt179 23860811

[5] Malik ZA Kott KS Poe AJ Kuo T Chen L Ferrara KW. Cardiac myocyte exosomes: stability, HSP60, and proteomics. Am J Physiol Heart Circ Physiol. (2013) 304:H954–65. 10.1152/ajpheart.00835.2012 23376832 PMC3625894

[6] Chistiakov DA Orekhov AN Bobryshev YV. Cardiac extracellular vesicles in normal and infarcted heart. Int J Mol Sci. (2016) 17:E63. 10.3390/ijms17010063 26742038 PMC4730308

[7] Yu X Deng L Wang D Li N Chen X Cheng X. Mechanism of TNF-α autocrine effects in hypoxic cardiomyocytes: initiated by hypoxia inducible factor 1alpha, presented by exosomes. J Mol Cell Cardiol. (2012) 53:848–57. 10.1016/j.yjmcc.2012.10.002 23085511

[8] Davidson MM Nesti C Palenzuela L Walker WF Hernandez E Protas L. Novel cell lines derived from adult human ventricular cardiomyocytes. J Mol Cell Cardiol. (2005) 39:133–47. 10.1016/j.yjmcc.2005.03.003 15913645

[9] Théry C Amigorena S Raposo G Clayton A Isolation and characterization of exosomes from cell culture supernatants and biological fluids. Curr Protoc Cell Biol. (2006) 30, 3.22.1–3.22.29. 10.1002/0471143030.cb0322s30 18228490

[10] Czernek L Chworos A Duechler M. The uptake of extracellular vesicles is affected by the differentiation status of myeloid cells. Scand J Immunol. (2015) 82:506–14. 10.1111/sji.12371 26332303

[11] Garcia NA Ontoria-Oviedo I Gonzalez-King H Diez-Juan A Sepulveda P. Glucose starvation in cardiomyocytes enhances exosome secretion and promotes angiogenesis in endothelial cells. PLoS ONE (2015) 10:e0138849. 10.1371/journal.pone.0138849 26393803 PMC4578916

[12] Gonzalez-King H Garcia NA Ontoria-Oviedo I Ciria M Montero JA Sepulveda P. Hypoxia inducible factor-1α potentiates jagged 1-mediated angiogenesis by mesenchymal stem cell-derived exosomes. Stem Cells (2017) 35:1747–59. 10.1002/stem.2618 28376567

[13] Liang CC Park AY Guan JL. *In vitro* scratch assay: a convenient and inexpensive method for analysis of cell migration *in vitro*. Nat Protoc. (2007) 2:329–33. 10.1038/nprot.2007.30 17406593

[14] Alonso R Salavert F Garcia-Garcia F Carbonell-Caballero J Bleda M Garcia-Alonso L Babelomics 5.0: functional interpretation for new generations of genomic data. Nucleic Acids Res. (2015) 43:W117–21. 10.1093/nar/gkv384 PMC4489263 25897133

[15] Al-Shahrour F Diaz-Uriarte R Dopazo J. FatiGO: a web tool for finding significant associations of Gene Ontology terms with groups of genes. Bioinformatics (2004) 20:578–80. 10.1093/bioinformatics/btg455 14990455

[16] Ashburner M Ball CA Blake JA Botstein D Butler H Cherry JM. Gene ontology: tool for the unification of biology. The Gene Ontology Consortium. Nat Genet. (2000) 25:25–9. 10.1038/75556 10802651 PMC3037419

[17] Supek F Bosnjak M Skunca N Smuc T. REVIGO summarizes and visualizes long lists of gene ontology terms. PLoS ONE (2011) 6:e21800. 10.1371/journal.pone.0021800 21789182 PMC3138752

[18] Loyer X Zlatanova I Devue C Yin M Howangyin KY Klaihmon P. Intra-cardiac release of extracellular vesicles shapes inflammation following myocardial infarction. Circ Res. (2018) 123:100–6. 10.1161/CIRCRESAHA.117.311326 29592957 PMC6023578

[19] Barile L Cervio E Lionetti V Milano G Ciullo A Biemmi V. Cardioprotection by cardiac progenitor cell-secreted exosomes: role of pregnancy-associated plasma protein-A. Cardiovasc Res. (2018) 114:992–1005. 10.1093/cvr/cvy055 29518183

[20] Sluijter JP Verhage V Deddens JC van den Akker F Doevendans PA. Microvesicles and exosomes for intracardiac communication. Cardiovasc Res. (2014) 102:302–11. 10.1093/cvr/cvu022 24488559

[21] Sluijter JPG Davidson SM Boulanger CM Buzas EI de Kleijn DPV Engel FB. Extracellular vesicles in diagnostics and therapy of the ischaemic heart: Position Paper from the Working Group on Cellular Biology of the Heart of the European Society of Cardiology. Cardiovasc Res. (2018) 114:19–34. 10.1093/cvr/cvx211 29106545 PMC5852624

[22] El Harane N Kervadec A Bellamy V Pidial L Neametalla HJ Perier MC Acellular therapeutic approach for heart failure: *in vitro* production of extracellular vesicles from human cardiovascular progenitors. Eur Heart J. (2018) 39:1835–47. 10.1093/eurheartj/ehy012 29420830 PMC6251654

[23] Naji DH Tan C Han F Zhao Y Wang J Wang D. Significant genetic association of a functional TFPI variant with circulating fibrinogen levels and coronary artery disease. Mol Genet Genomics (2018) 293:119–28. 10.1007/s00438-017-1365-6 28894953 PMC5794607

[24] Ayre DC Chute IC Joy AP Barnett DA Hogan AM Grull MP. CD24 induces changes to the surface receptors of B cell microvesicles with variable effects on their RNA and protein cargo. Sci Rep. (2017) 7:8642. 10.1038/s41598-017-08094-8 28819186 PMC5561059

[25] Wang X Huang W Liu G Cai W Millard RW Wang Y. Cardiomyocytes mediate anti-angiogenesis in type 2 diabetic rats through the exosomal transfer of miR-320 into endothelial cells. J Mol Cell Cardiol. (2014) 74:139–50. 10.1016/j.yjmcc.2014.05.001 24825548 PMC4120246

[26] Ribeiro-Rodrigues TM Laundos TL Pereira-Carvalho R Batista-Almeida D Pereira R Coelho-Santos V. Exosomes secreted by cardiomyocytes subjected to ischaemia promote cardiac angiogenesis. Cardiovasc Res. (2017) 113:1338–50. 10.1093/cvr/cvx118 28859292

[27] Hermida N Michel L Esfahani H Dubois-Deruy E Hammond J Bouzin C. Cardiac myocyte beta3-adrenergic receptors prevent myocardial fibrosis by modulating oxidant stress-dependent paracrine signaling. Eur Heart J. (2018) 39: 888–98. 10.1093/eurheartj/ehx366 29106524

[28] Chaturvedi P Kalani A Medina I Familtseva A Tyagi SC. Cardiosome mediated regulation of MMP9 in diabetic heart: role of mir29b and mir455 in exercise. J Cell Mol Med. (2015) 19:2153–61. 10.1111/jcmm.12589 25824442 PMC4568920

