# Supplementary figures and images for: Extracellular Vesicles Secreted by Hypoxic AC10 Cardiomyocytes Modulate Fibroblast Cell Motility
Source: Front Cardiovasc Med. 2018 Oct 25;5:152. doi: 10.3389/fcvm.2018.00152 (PMC6209632; doi:10.3389/fcvm.2018.00152)

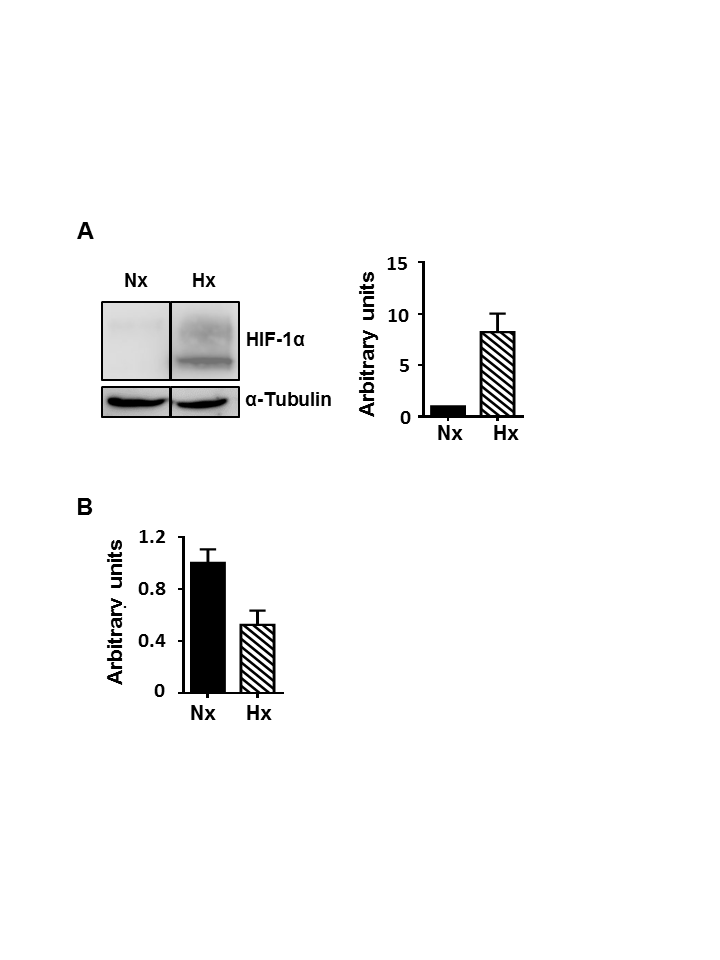

Supplement: Figure S1 — (A) Representative western blot images of proteins extracted from AC10 cell cultures in normoxia or hypoxia (2% O2). Lanes were loaded with equal amounts of protein and incubated with a hypoxia inducible factor-1 antibody. Signals were quantified by densitometry (n = 3). (B) Quantification of viable AC10 by CCK8 after different culture conditions used to isolate EVs. [file Image_1.tif]

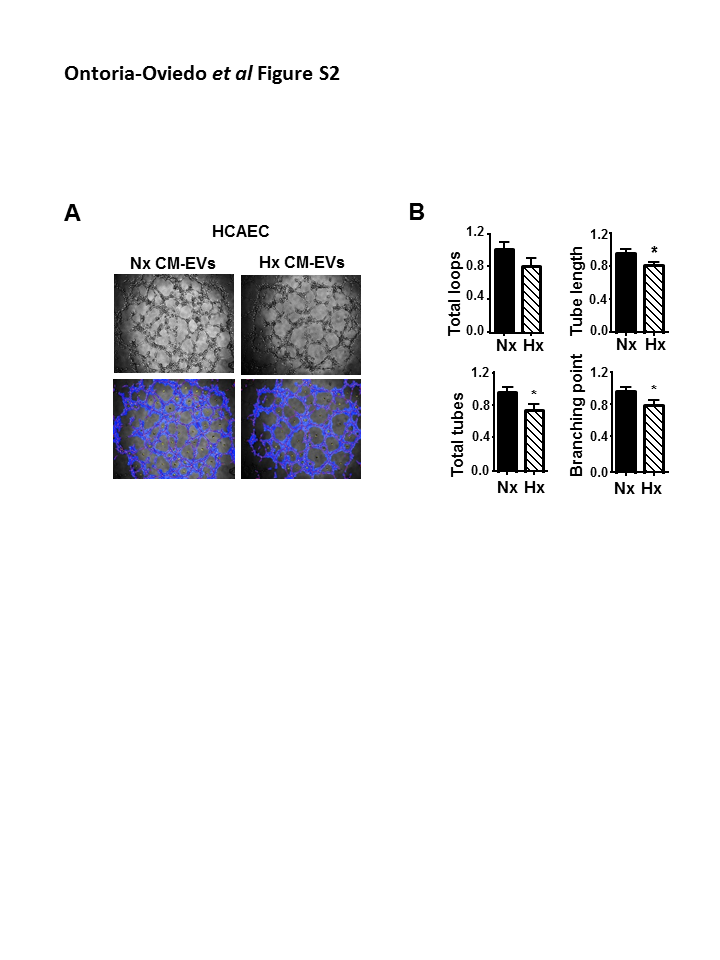

Supplement: Figure S2 — Tube formation assay of human coronary artery endothelial cells (HCAEC). (A) Representative images of tube formation after 6 h of culture in the presence of normoxia (Nx) or hypoxia (Hx) cardiomyocyte-derived extracellular vesicles. (B) Quantification of total loops, tube length, total tubes and number of branching points from images taken after 6 h culture. Results are expressed in arbitrary units (n = 3, *P < 0.05). [file Image_2.tif]

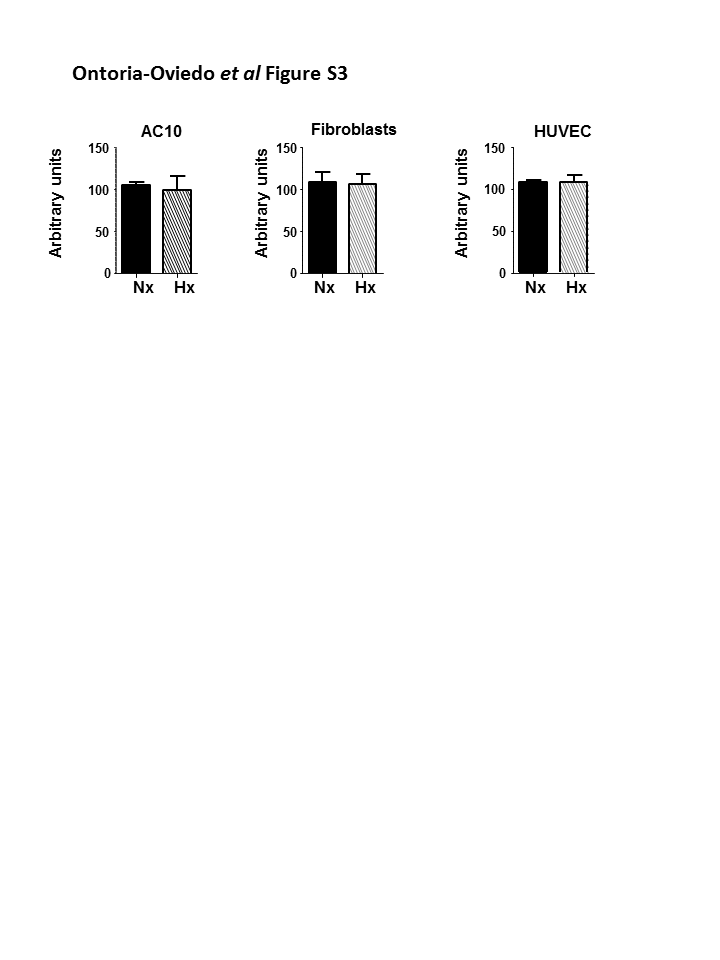

Supplement: Figure S3 — Cell proliferation assay of AC10, fibroblasts and human umbilical cord vein cells (HUVEC). Representative example of cell proliferation measured by CCK-8 assay after 48 h of culture in presence of Nx and Hx CM-EVs. Results are presented in arbitrary units (n = 3). [file Image_3.tif]
