# Supplementary material for: Extracellular Vesicles Secreted by Hypoxic AC10 Cardiomyocytes Modulate Fibroblast Cell Motility
Source: Front Cardiovasc Med. 2018 Oct 25;5:152. doi: 10.3389/fcvm.2018.00152 (PMC6209632; doi:10.3389/fcvm.2018.00152)
Supplement: Table S1 — Protein identification in cardiomyocyte-derived extracellular vesicles in normoxia. [file Table_1.DOCX]

**Tables**

**Table S1**-Protein identification in cardiomyocyte-derived extracellular vesicles in normoxia.

| Unused | %Cov | Accession | Name | Peptides (95%) |
| --- | --- | --- | --- | --- |
| 71.89 | 47.13 | sp\|Q92626\|PXDN_HUMAN | Peroxidasin homolog | 46 |
| 60.32 | 23.41 | sp\|Q99715\|COCA1_HUMAN | Collagen alpha-1(XII) chain | 32 |
| 59.83 | 23.95 | sp\|P12111\|CO6A3_HUMAN | Collagen alpha-3(VI) chain | 32 |
| 42.34 | 25.73 | sp\|P02751\|FINC_HUMAN | Fibronectin | 22 |
| 39.69 | 79.07 | sp\|Q08431\|MFGM_HUMAN | Lactadherin | 32 |
| 38.55 | 31.34 | sp\|Q00610\|CLH1_HUMAN | Clathrin heavy chain 1 | 21 |
| 38.15 | 29.32 | sp\|P07996\|TSP1_HUMAN | Thrombospondin-1 | 26 |
| 31.69 | 47.00 | sp\|Q15582\|BGH3_HUMAN | Transforming growth factor-beta-induced protein ig-h3 | 21 |
| 27.74 | 40.85 | sp\|Q08380\|LG3BP_HUMAN | Galectin-3-binding protein | 15 |
| 26.74 | 25.00 | sp\|P35442\|TSP2_HUMAN | Thrombospondin-2 | 15 |
| 21.89 | 13.18 | sp\|Q6UVK1\|CSPG4_HUMAN | Chondroitin sulfate proteoglycan 4 | 13 |
| 19.84 | 6.79 | sp\|P98160\|PGBM_HUMAN | Basement membrane-specific heparan sulfate proteoglycan core protein | 12 |
| 18.07 | 41.43 | sp\|P14618\|KPYM_HUMAN | Pyruvate kinase PKM | 10 |
| 17.76 | 43.65 | sp\|Q99880\|H2B1L_HUMAN | Histone H2B type 1-L | 24 |
| 13.24 | 9.87 | sp\|O00468\|AGRIN_HUMAN | Agrin | 7 |
| 12.07 | 23.53 | sp\|Q9UKU9\|ANGL2_HUMAN | Angiopoietin-related protein 2 | 7 |
| 11.72 | 21.05 | sp\|P05556\|ITB1_HUMAN | Integrin beta-1 | 9 |
| 11.29 | 15.47 | sp\|P12109\|CO6A1_HUMAN | Collagen alpha-1(VI) | 8 |
| 11.02 | 17.13 | sp\|Q969P0\|IGSF8_HUMAN | Immunoglobulin superfamily member 8 | 7 |
| 10.37 | 45.64 | sp\|O00560\|SDCB1_HUMAN | Syntenin-1 | 8 |
| 10.28 | 16.24 | sp\|Q9Y6C2\|EMIL1_HUMAN | EMILIN-1 | 7 |
| 10.06 | 14.03 | sp\|P14543\|NID1_HUMAN | Nidogen-1 | 8 |
| 9.47 | 13.84 | sp\|P12110\|CO6A2_HUMAN | Collagen alpha-2(VI) chain | 5 |
| 8.99 | 25.84 | sp\|P10909\|CLUS_HUMAN | Clusterin | 5 |
| 8.11 | 22.41 | sp\|Q9P2B2\|FPRP_HUMAN | Prostaglandin F2 receptor negative regulator | 5 |
| 8.05 | 22.10 | sp\|P30495\|1B56_HUMAN | HLA class I histocompatibility antigen. B-56 alpha chain | 5 |
| 7.92 | 15.89 | sp\|Q76M96\|CCD80_HUMAN | Coiled-coil domain-containing protein 80 | 5 |
| 7.84 | 6.14 | sp\|O15230\|LAMA5_HUMAN | Laminin subunit alpha-5 | 5 |
| 7.74 | 7.04 | sp\|P01031\|CO5_HUMAN | Complement C5 | 4 |
| 5.91 | 12.94 | sp\|P26006\|ITA3_HUMAN | Integrin alpha-3 | 3 |
| 5.76 | 24.84 | sp\|P04216\|THY1_HUMAN | Thy-1 membrane glycoprotein | 4 |
| 4.49 | 2.56 | sp\|P04114\|APOB_HUMAN | Apolipoprotein B-100 | 3 |
| 4.19 | 11.66 | sp\|Q9C0H2\|TTYH3_HUMAN | Protein tweety homolog 3 | 2 |
| 4.01 | 3.78 | sp\|P13612\|ITA4_HUMAN | Integrin alpha-4 | 2 |
| 2.68 | 3.12 | sp\|Q8IZ83\|A16A1_HUMAN | Aldehyde dehydrogenase family 16 member | 2 |
| 2.28 | 3.27 | sp\|P11387\|TOP1_HUMAN | DNA topoisomerase 1 | 1 |
| 2.27 | 16.26 | sp\|Q15113\|PCOC1_HUMAN | Procollagen C-endopeptidase enhancer 1 | 1 |
| 2.20 | 7.11 | sp\|Q9Y4K0\|LOXL2_HUMAN | Lysyl oxidase homolog 2 | 2 |
| 2.04 | 7.18 | sp\|Q13822\|ENPP2_HUMAN | Ectonucleotide pyrophosphatase/phosphodiesterase family member 2 | 1 |
| 2.03 | 20.17 | sp\|O14817\|TSN4_HUMAN | Tetraspanin-4 | 2 |
| 2.00 | 13.51 | sp\|Q9UK55\|ZPI_HUMAN | Protein Z-dependent protease inhibitor | 1 |
| 2.00 | 5.12 | sp\|P40189\|IL6RB_HUMAN | Interleukin-6 receptor subunit beta | 1 |
| 2.00 | 6.93 | sp\|Q16769\|QPCT_HUMAN | Glutaminyl-peptide cyclotransferase | 1 |
| 2.00 | 4.18 | sp\|P21589\|5NTD_HUMAN | 5'-nucleotidase | 1 |
| 2.00 | 1.52 | sp\|P98095\|FBLN2_HUMAN | Fibulin-2 | 1 |
| 2.00 | 11.40 | sp\|P06702\|S10A9_HUMAN | Protein S100-A9 | 1 |
| 1.95 | 1.69 | sp\|Q9UBG0\|MRC2_HUMAN | C-type mannose receptor 2 | 1 |
| 1.83 | 5.44 | sp\|Q9Y5C1\|ANGL3_HUMAN | Angiopoietin-related protein 3 | 2 |
| 1.81 | 8.67 | sp\|Q9Y240\|CLC11_HUMAN | C-type lectin domain family 11 member A | 2 |
| 1.68 | 3.56 | sp\|Q99536\|VAT1_HUMAN | Synaptic vesicle membrane protein VAT-1 homolog | 1 |
| 1.62 | 9.65 | sp\|P21926\|CD9_HUMAN | CD9 antigen | 1 |
| 1.39 | 1.22 | sp\|O00159\|MYO1C_HUMAN | Unconventional myosin-Ic | 1 |
| 1.38 | 23.44 | sp\|Q71UI9\|H2AV_HUMAN | Histone H2A.V | 3 |
