# Supplementary material for: Extracellular Vesicles Secreted by Hypoxic AC10 Cardiomyocytes Modulate Fibroblast Cell Motility
Source: Front Cardiovasc Med. 2018 Oct 25;5:152. doi: 10.3389/fcvm.2018.00152 (PMC6209632; doi:10.3389/fcvm.2018.00152)
Supplement: Table S2 — Protein identification in cardiomyocyte-derived extracellular vesicles in hypoxia. [file Table_2.DOCX]

**Tables**

**Table S2**-Protein identification in cardiomyocyte-derived extracellular vesicles in hypoxia.

| Unused | %Cov | Accession | Name | Peptides (95%) |
| --- | --- | --- | --- | --- |
| 195.57 | 53.23 | sp\|P12111\|CO6A3_HUMAN | Collagen alpha-3(VI) chain | 109 |
| 125.36 | 38.10 | sp\|Q99715\|COCA1_HUMAN | Collagen alpha-1(XII) chain | 67 |
| 102.35 | 29.24 | sp\|P98160\|PGBM_HUMAN | Basement membrane-specific heparan sulfate proteoglycan core protein | 57 |
| 86.44 | 38.39 | sp\|P02751\|FINC_HUMAN | Fibronectin | 45 |
| 63.61 | 43.70 | sp\|Q00610\|CLH1_HUMAN | Clathrin heavy chain | 35 |
| 54.75 | 40.97 | sp\|Q92626\|PXDN_HUMAN | Peroxidasin homolog | 32 |
| 54.40 | 40.68 | sp\|P07996\|TSP1_HUMAN | Thrombospondin-1 | 33 |
| 46.41 | 61.64 | sp\|Q15582\|BGH3_HUMAN | Transforming growth factor-beta-induced protein ig-h3 | 29 |
| 43.34 | 52.99 | sp\|Q08380\|LG3BP_HUMAN | Galectin-3-binding protein | 28 |
| 41.31 | 40.47 | sp\|P12109\|CO6A1_HUMAN | Collagen alpha-1(VI) chain | 27 |
| 40.64 | 37.59 | sp\|P12110\|CO6A2_HUMAN | Collagen alpha-2(VI) chain | 25 |
| 37.04 | 30.38 | sp\|P35442\|TSP2_HUMAN | Thrombospondin-2 | 21 |
| 36.33 | 21.72 | sp\|O00468\|AGRIN_HUMAN | Agrin | 20 |
| 31.4 | 69.77 | sp\|Q08431\|MFGM_HUMAN | Lactadherin | 22 |
| 29.86 | 23.80 | sp\|P11047\|LAMC1_HUMAN | Laminin subunit gamma-1 | 15 |
| 29.84 | 17.23 | sp\|Q6UVK1\|CSPG4_HUMAN | Chondroitin sulfate proteoglycan 4 | 16 |
| 27.94 | 47.80 | sp\|Q9Y4K0\|LOXL2_HUMAN | Lysyl oxidase homolog 2 | 16 |
| 26.22 | 27.27 | sp\|P14543\|NID1_HUMAN | Nidogen-1 | 14 |
| 25.29 | 57.69 | sp\|Q8IUE6\|H2A2B_HUMAN | Histone H2A type 2-B | 19 |
| 25.20 | 15.92 | sp\|P35555\|FBN1_HUMAN | Fibrillin-1 | 12 |
| 22.20 | 26.07 | sp\|P05556\|ITB1_HUMAN | Integrin beta-1 | 12 |
| 21.35 | 10.42 | sp\|O15230\|LAMA5_HUMAN | Laminin subunit alpha-5 | 12 |
| 19.96 | 21.06 | sp\|Q9Y6C2\|EMIL1_HUMAN | EMILIN-1 | 12 |
| 19.70 | 36.39 | sp\|Q14764\|MVP_HUMAN | Major vault protein | 12 |
| 19.19 | 43.88 | sp\|P14618\|KPYM_HUMAN | Pyruvate kinase PKM | 12 |
| 16.87 | 14.61 | sp\|P07942\|LAMB1_HUMAN | Laminin subunit beta-1 | 10 |
| 16.52 | 43.01 | sp\|P01892\|1A02_HUMAN | HLA class I histocompatibility antigen. A-2 alpha chain | 9 |
| 15.80 | 15.53 | sp\|P53396\|ACLY_HUMAN | ATP-citrate synthase | 11 |
| 15.53 | 45.84 | sp\|Q9UKU9\|ANGL2_HUMAN | Angiopoietin-related protein 2 | 10 |
| 15.51 | 49.21 | sp\|Q99880\|H2B1L_HUMAN | Histone H2B type 1-L | 27 |
| 14.94 | 61.07 | sp\|O00560\|SDCB1_HUMAN | Syntenin-1 | 10 |
| 14.15 | 11.07 | sp\|P49327\|FAS_HUMAN | Fatty acid synthase | 7 |
| 13.69 | 7.48 | sp\|Q07954\|LRP1_HUMAN | Prolow-density lipoprotein receptor-related protein 1 | 9 |
| 13.39 | 31.74 | sp\|P49368\|TCPG_HUMAN | T-complex protein 1 subunit gamma | 7 |
| 12.86 | 58.27 | sp\|O94907\|DKK1_HUMAN | Dickkopf-related protein 1 | 8 |
| 12.1 | 23.47 | sp\|P16403\|H12_HUMAN | Histone H1.2 | 6 |
| 11.6 | 34.81 | sp\|P12956\|XRCC6_HUMAN | X-ray repair cross-complementing protein 6 | 7 |
| 11.02 | 18.44 | sp\|P13010\|XRCC5_HUMAN | X-ray repair cross-complementing protein 5 | 6 |
| 10.83 | 16.27 | sp\|Q9P2B2\|FPRP_HUMAN | Prostaglandin F2 receptor negative regulator | 7 |
| 10.1 | 34.71 | sp\|P17987\|TCPA_HUMAN | T-complex protein 1 subunit alpha | 6 |
| 9.89 | 23.25 | sp\|P21926\|CD9_HUMAN | CD9 antigen OS=Homo sapiens | 5 |
| 9.17 | 38.12 | sp\|P30488\|1B50_HUMAN | HLA class I histocompatibility antigen. B-50 alpha chain | 8 |
| 9.08 | 16.48 | sp\|P10909\|CLUS_HUMAN | Clusterin | 5 |
| 9.07 | 13.09 | sp\|P23142\|FBLN1_HUMAN | Fibulin-1 | 5 |
| 8.00 | 24.84 | sp\|P04216\|THY1_HUMAN | Thy-1 membrane glycoprotein | 4 |
| 7.60 | 8.95 | sp\|P21333\|FLNA_HUMAN | Filamin-A | 7 |
| 7.59 | 12.29 | sp\|Q16363\|LAMA4_HUMAN | Laminin subunit alpha-4 | 5 |
| 7.47 | 23.33 | sp\|Q969P0\|IGSF8_HUMAN | Immunoglobulin superfamily member 8 | 5 |
| 6.44 | 9.43 | sp\|P01031\|CO5_HUMAN | Complement C5 | 4 |
| 6.25 | 12.27 | sp\|P26006\|ITA3_HUMAN | Integrin alpha-3 | 4 |
| 6.00 | 10.77 | sp\|Q15043\|S39AE_HUMAN | Zinc transporter ZIP14 | 3 |
| 5.62 | 12.69 | sp\|P04278\|SHBG_HUMAN | Sex hormone-binding globulin | 3 |
| 5.27 | 11.78 | sp\|Q9HB63\|NET4_HUMAN | Netrin-4 | 3 |
| 5.12 | 10.30 | sp\|P02461\|CO3A1_HUMAN | Collagen alpha-1(III) chain | 3 |
| 5.02 | 17.59 | sp\|P26022\|PTX3_HUMAN | Pentraxin-related protein | 3 |
| 4.74 | 15.71 | sp\|P08195\|4F2_HUMAN | 4F2 cell-surface antigen heavy | 3 |
| 4.53 | 18.60 | sp\|Q99832\|TCPH_HUMAN | T-complex protein 1 subunit beta | 3 |
| 4.35 | 17.11 | sp\|P10646\|TFPI1_HUMAN | Tissue factor pathway inhibitor | 3 |
| 4.34 | 20.56 | sp\|P21589\|5NTD_HUMAN | 5'-nucleotidase | 3 |
| 4.14 | 9.75 | sp\|Q9C0H2\|TTYH3_HUMAN | Protein tweety homolog 3 | 2 |
| 4.11 | 8.62 | sp\|P13612\|ITA4_HUMAN | Integrin alpha-4 | 2 |
| 4.00 | 6.33 | sp\|Q02809\|PLOD1_HUMAN | Procollagen-lysine.2-oxoglutarate 5-dioxygenase 1 | 2 |
| 3.99 | 12.63 | sp\|P08133\|ANXA6_HUMAN | Annexin A6 | 2 |
| 3.94 | 22.49 | sp\|Q15113\|PCOC1_HUMAN | Procollagen C-endopeptidase enhancer 1 | 2 |
| 3.73 | 6.10 | sp\|Q15758\|AAAT_HUMAN | Neutral amino acid transporter B(0) | 2 |
| 3.51 | 5.75 | sp\|Q14112\|NID2_HUMAN | Nidogen-2 | 2 |
| 3.45 | 44.78 | sp\|P04406\|G3P_HUMAN | Glyceraldehyde-3-phosphate dehydrogenase | 7 |
| 3.43 | 11.15 | sp\|Q9Y240\|CLC11_HUMAN | C-type lectin domain family 11 member A | 2 |
| 3.41 | 9.63 | sp\|O14672\|ADA10_HUMAN | Disintegrin and metalloproteinase domain-containing protein 10 | 2 |
| 3.21 | 18.92 | sp\|P02452\|CO1A1_HUMAN | Collagen alpha-1(I) chain | 11 |
| 3.13 | 20.59 | sp\|O14817\|TSN4_HUMAN | Tetraspanin-4 | 2 |
| 3.01 | 8.07 | sp\|Q8IWA5\|CTL2_HUMAN | Choline transporter-like protein 2 | 2 |
| 2.98 | 11.92 | sp\|P49746\|TSP3_HUMAN | Thrombospondin-3 | 2 |
| 2.77 | 11.02 | sp\|Q9Y230\|RUVB2_HUMAN | RuvB-like 2 | 3 |
| 2.69 | 10.88 | sp\|O75954\|TSN9_HUMAN | Tetraspanin-9 | 2 |
| 2.32 | 7.20 | sp\|P13497\|BMP1_HUMAN | Bone morphogenetic protein 1 | 2 |
| 2.29 | 4.56 | sp\|Q14204\|DYHC1_HUMAN | Cytoplasmic dynein 1 heavy chain 1 | 1 |
| 2.23 | 8.00 | sp\|Q9UGM3\|DMBT1_HUMAN | Deleted in malignant brain tumors 1 protein | 1 |
| 2.09 | 23.66 | sp\|P48061\|SDF1_HUMAN | Stromal cell-derived factor 1 | 1 |
| 2.06 | 4.18 | sp\|P16070\|CD44_HUMAN | CD44 antigen | 1 |
| 2.05 | 10.85 | sp\|Q8IZ83\|A16A1_HUMAN | Aldehyde dehydrogenase family 16 member A1 | 1 |
| 2.00 | 20.28 | sp\|P01023\|A2MG_HUMAN | Alpha-2-macroglobulin | 32 |
| 2.00 | 9.30 | sp\|P50914\|RL14_HUMAN | Mas-related G-protein coupled receptor member F | 1 |
| 2.00 | 1.83 | sp\|P08514\|ITA2B_HUMAN | Integrin alpha-IIb | 1 |
| 2.00 | 4.08 | sp\|Q96AM1\|MRGRF_HUMAN | Mas-related G-protein coupled receptor member F | 1 |
| 2.00 | 15.48 | sp\|Q71UM5\|RS27L_HUMAN | 40S ribosomal protein S27-like | 1 |
| 1.80 | 3.83 | sp\|Q86YZ3\|HORN_HUMAN | Hornerin | 1 |
| 1.80 | 5.62 | sp\|Q13443\|ADAM9_HUMAN | Disintegrin and metalloproteinase domain-containing protein 9 | 1 |
| 1.74 | 39.45 | sp\|P01891\|1A68_HUMAN | HLA class I histocompatibility antigen. A-68 alpha chain | 8 |
| 1.74 | 5.94 | sp\|Q14974\|IMB1_HUMAN | Importin subunit beta-1 | 1 |
| 1.72 | 5.08 | sp\|P05106\|ITB3_HUMAN | Integrin beta-3 | 1 |
| 1.70 | 5.53 | sp\|O14786\|NRP1_HUMAN | Neuropilin-1 | 1 |
| 1.70 | 3.19 | sp\|Q9UNM6\|PSD13_HUMAN | 26S proteasome non-ATPase regulatory subunit 13 | 1 |
| 1.62 | 5.38 | sp\|P15144\|AMPN_HUMAN | Aminopeptidase N | 1 |
| 1.57 | 3.70 | sp\|Q9NRN5\|OLFL3_HUMAN | Olfactomedin-like protein 3 | 1 |
| 1.55 | 4.60 | sp\|P28072\|PSB6_HUMAN | Proteasome subunit beta type-6 | 1 |
| 1.50 | 10.07 | sp\|P26641\|EF1G_HUMAN | Elongation factor 1-gamma | 2 |
| 1.46 | 2.27 | sp\|P54289\|CA2D1_HUMAN | Voltage-dependent calcium channel subunit alpha-2/delta-1 | 1 |
| 1.33 | 9.95 | sp\|P26373\|RL13_HUMAN | 60S ribosomal protein L13 | 1 |
