# Supplementary material for: Extracellular Vesicles Secreted by Hypoxic AC10 Cardiomyocytes Modulate Fibroblast Cell Motility
Source: Front Cardiovasc Med. 2018 Oct 25;5:152. doi: 10.3389/fcvm.2018.00152 (PMC6209632; doi:10.3389/fcvm.2018.00152)
Supplement: Table S3 — Gene ontology biological processes for proteins identified in extracellular vesicles derived in normoxia. [file Table_3.DOCX]

**Tables**

**Table S3**- Gene ontology biological processes for proteins identified in extracellular vesicles derived in normoxia.

| GO_id | Term | p-value |
| --- | --- | --- |
| GO:0070062 | extracellular vesicular exosome | 3.79E-46 |
| GO:0005576 | extracellular region | 2.28E-37 |
| GO:0005615 | extracellular space | 3.19E-37 |
| GO:0031012 | extracellular matrix | 2.61E-34 |
| GO:0030198 | extracellular matrix organization | 2.99E-29 |
| GO:0005578 | proteinaceous extracellular matrix | 7.44E-24 |
| GO:0005604 | basement membrane | 2.53E-21 |
| GO:0005178 | integrin binding | 3.21E-19 |
| GO:0007155 | cell adhesion | 1.32E-16 |
| GO:0005788 | endoplasmic reticulum lumen | 3.13E-10 |
| GO:0007160 | cell-matrix adhesion | 3.54E-10 |
| GO:0005925 | focal adhesion | 4.12E-10 |
| GO:0007229 | integrin-mediated signaling pathway | 6.65E-10 |
| GO:0002576 | platelet degranulation | 7.89E-10 |
| GO:0005518 | collagen binding | 9.41E-09 |
| GO:0009986 | cell Surface | 1.39E-08 |
| GO:1903561 | extracellular vesicle | 1.24E-07 |
| GO:0005201 | extracellular matrix structural constituent | 2.99E-07 |
| GO:0010811 | positive regulation of cell-substrate adhesion | 6.38E-07 |
| GO:0044267 | cellular protein metabolic process | 1.21E-06 |
| GO:0001968 | fibronectin binding | 1.80E-06 |
| GO:0022617 | extracellular matrix disassembly | 3.02E-06 |
| GO:0030574 | collagen catabolic process | 3.53E-06 |
| GO:0035987 | endodermal cell differentiation | 6.10E-06 |
| GO:0010951 | negative regulation of endopeptidase activity | 7.96E-06 |
| GO:0070268 | Cornification | 9.65E-06 |
| GO:0008201 | heparin binding | 1.21E-05 |
| GO:0043236 | laminin binding | 1.28E-05 |
| GO:0034446 | substrate adhesion-dependent cell spreading | 2.37E-05 |
| GO:0043312 | neutrophil degranulation | 4.29E-05 |
| GO:0031424 | Keratinization | 4.29E-05 |
| GO:0005198 | structural molecule activity | 5.29E-05 |
| GO:0007044 | cell-substrate junction assembly | 5.79E-05 |
| GO:0072562 | blood microparticle | 7.02E-05 |
| GO:0005581 | collagen trimer | 7.88E-05 |
| GO:0031093 | platelet alpha granule lumen | 8.43E-05 |
| GO:0033627 | cell adhesion mediated by integrin | 1.13E-04 |
| GO:0004866 | endopeptidase inhibitor activity | 1.41E-04 |
| GO:0048407 | platelet-derived growth factor binding | 2.28E-04 |
| GO:0050840 | extracellular matrix binding | 2.31E-04 |
| GO:0019838 | growth factor binding | 2.89E-04 |
| GO:0005882 | intermediate filament | 2.89E-04 |
| GO:0001525 | Angiogenesis | 3.38E-04 |
| GO:0010466 | negative regulation of peptidase activity | 3.46E-04 |
| GO:0005200 | structural constituent of cytoskeleton | 3.76E-04 |
| GO:0043259 | laminin-10 complex | 3.86E-04 |
| GO:0018149 | peptide cross-linking | 4.56E-04 |
| GO:0002020 | protease binding | 5.32E-04 |
| GO:0005509 | calcium ion binding | 5.32E-04 |
| GO:0004867 | serine-type endopeptidase inhibitor activity | 5.32E-04 |
| GO:0000786 | Nucleosome | 5.71E-04 |
| GO:0050731 | positive regulation of peptidyl-tyrosine phosphorylation | 7.24E-04 |
| GO:0031581 | hemidesmosome assembly | 9.01E-04 |
| GO:0045095 | keratin filament | 1.10E-03 |
| GO:0008305 | integrin complex | 1.33E-03 |
| GO:0071062 | alphav-beta3 integrin-vitronectin complex | 1.54E-03 |
| GO:0033631 | cell-cell adhesion mediated by integrin | 1.54E-03 |
| GO:0005044 | scavenger receptor activity | 2.03E-03 |
| GO:0050900 | leukocyte migration | 2.24E-03 |
| GO:0006898 | receptor-mediated endocytosis | 2.24E-03 |
| GO:0030414 | peptidase inhibitor activity | 2.35E-03 |
| GO:0030335 | positive regulation of cell migration | 2.40E-03 |
| GO:0045766 | positive regulation of angiogenesis | 2.43E-03 |
| GO:0019960 | C-X3-C chemokine binding | 2.43E-03 |
| GO:0016477 | cell migration | 2.60E-03 |
| GO:0030199 | collagen fibril organization | 7.05E-03 |
| GO:0071711 | basement membrane organization | 7.73E-03 |
| GO:0034113 | heterotypic cell-cell adhesion | 8.59E-03 |
| GO:0005577 | fibrinogen complex | 1.01E-02 |
| GO:0034667 | integrin alpha3-beta1 complex | 1.01E-02 |
| GO:0050750 | low-density lipoprotein particle receptor binding | 1.23E-02 |
| GO:0001867 | complement activation, lectin pathway | 1.31E-02 |
| GO:0005614 | interstitial matrix | 1.64E-02 |
| GO:0033622 | integrin activation | 1.64E-02 |
| GO:0007159 | leukocyte cell-cell adhesion | 2.06E-02 |
| GO:0030280 | structural constituent of epidermis | 2.06E-02 |
| GO:0070208 | protein heterotrimerization | 2.49E-02 |
| GO:0030449 | regulation of complement activation | 2.51E-02 |
| GO:0007599 | Hemostasis | 2.52E-02 |
| GO:0030023 | extracellular matrix constituent conferring elasticity | 2.52E-02 |
| GO:0070051 | fibrinogen binding | 2.52E-02 |
| GO:0004556 | alpha-amylase activity | 2.52E-02 |
| GO:0103025 | alpha-amylase activity (releasing maltohexaose) | 2.52E-02 |
| GO:0035606 | peptidyl-cysteine S-trans-nitrosylation | 2.52E-02 |
| GO:0030168 | platelet activation | 2.69E-02 |
| GO:0016504 | peptidase activator activity | 2.73E-02 |
| GO:0042470 | Melanosome | 2.92E-02 |
| GO:0043687 | post-translational protein modification | 3.08E-02 |
| GO:0043394 | proteoglycan binding | 3.19E-02 |
| GO:0015026 | coreceptor activity | 3.65E-02 |
| GO:0030194 | positive regulation of blood coagulation | 3.65E-02 |
| GO:0010952 | positive regulation of peptidase activity | 3.65E-02 |
| GO:0048333 | mesodermal cell differentiation | 3.65E-02 |
| GO:0035578 | azurophil granule lumen | 3.69E-02 |
| GO:0009897 | external side of plasma membrane | 4.19E-02 |
| GO:0043260 | laminin-11 complex | 4.26E-02 |
| GO:0005606 | laminin-1 complex | 4.26E-02 |
| GO:0007161 | calcium-independent cell-matrix adhesion | 4.26E-02 |
| GO:0043564 | Ku70:Ku80 complex | 4.26E-02 |
| GO:0008544 | epidermis development | 4.59E-02 |
| GO:0005605 | basal lamina | 4.65E-02 |
