# Supplementary material for: Extracellular Vesicles Secreted by Hypoxic AC10 Cardiomyocytes Modulate Fibroblast Cell Motility
Source: Front Cardiovasc Med. 2018 Oct 25;5:152. doi: 10.3389/fcvm.2018.00152 (PMC6209632; doi:10.3389/fcvm.2018.00152)
Supplement: Table S4 — Gene ontology biological processes for proteins identified in extracellular vesicles derived in hypoxia. [file Table_4.DOCX]

**Tables**

**Table S4**- Gene ontology biological processes for proteins identified in extracellular vesicles derived in hypoxia.

| GO_id | Term | p-value |
| --- | --- | --- |
| GO:0070062 | extracellular vesicular exosome | 4.64E-50 |
| GO:0030198 | extracellular matrix organization | 9.94E-24 |
| GO:0005576 | extracellular region | 1.10E-23 |
| GO:0005615 | extracellular space | 1.63E-23 |
| GO:0031012 | extracellular matrix | 1.86E-22 |
| GO:0005578 | proteinaceous extracellular matrix | 7.31E-15 |
| GO:0007155 | cell adhesion | 4.76E-14 |
| GO:0005604 | basement membrane | 7.55E-14 |
| GO:0005178 | integrin binding | 1.72E-12 |
| GO:0005925 | focal adhesion | 9.02E-12 |
| GO:0009986 | cell surface | 1.22E-09 |
| GO:0070268 | cornification | 2.24E-09 |
| GO:0007229 | integrin-mediated signaling pathway | 6.20E-09 |
| GO:0002576 | platelet degranulation | 8.46E-09 |
| GO:0005198 | structural molecule activity | 2.57E-08 |
| GO:0007160 | cell-matrix adhesion | 3.70E-08 |
| GO:0031424 | keratinization | 3.94E-08 |
| GO:0043312 | neutrophil degranulation | 9.05E-08 |
| GO:0005200 | structural constituent of cytoskeleton | 2.61E-07 |
| GO:0005882 | intermediate filament | 3.58E-07 |
| GO:1903561 | extracellular vesicle | 4.95E-07 |
| GO:0005518 | collagen binding | 7.61E-07 |
| GO:0001968 | fibronectin binding | 5.12E-06 |
| GO:1904874 | positive regulation of telomerase RNA localization to Cajal body | 8.10E-06 |
| GO:0008305 | integrin complex | 8.67E-06 |
| GO:0022617 | extracellular matrix disassembly | 1.22E-05 |
| GO:0005201 | extracellular matrix structural constituent | 1.68E-05 |
| GO:0045296 | cadherin binding | 3.91E-05 |
| GO:0010811 | positive regulation of cell-substrate adhesion | 4.09E-05 |
| GO:0008201 | heparin binding | 6.62E-05 |
| GO:0034446 | substrate adhesion-dependent cell spreading | 6.78E-05 |
| GO:0017101 | aminoacyl-tRNA synthetase multienzyme complex | 7.09E-05 |
| GO:0018149 | peptide cross-linking | 8.33E-05 |
| GO:0007044 | cell-substrate junction assembly | 1.08E-04 |
| GO:0070051 | fibrinogen binding | 2.08E-04 |
| GO:0031093 | platelet alpha granule lumen | 2.92E-04 |
| GO:0035987 | endodermal cell differentiation | 3.26E-04 |
| GO:0045095 | keratin filament | 3.80E-04 |
| GO:0045109 | intermediate filament organization | 3.83E-04 |
| GO:0050840 | extracellular matrix binding | 5.99E-04 |
| GO:0043259 | laminin-10 complex | 7.07E-04 |
| GO:0043236 | laminin binding | 8.02E-04 |
| GO:1904871 | positive regulation of protein localization to Cajal body | 8.48E-04 |
| GO:0005788 | endoplasmic reticulum lumen | 1.02E-03 |
| GO:1904813 | ficolin-1-rich granule lumen | 1.03E-03 |
| GO:0002199 | zona pellucida receptor complex | 1.10E-03 |
| GO:0030574 | collagen catabolic process | 1.57E-03 |
| GO:0003723 | RNA binding | 1.66E-03 |
| GO:0001525 | angiogenesis | 1.82E-03 |
| GO:0072562 | blood microparticle | 1.82E-03 |
| GO:0005832 | chaperonin-containing T-complex | 1.82E-03 |
| GO:0002020 | protease binding | 1.95E-03 |
| GO:0042470 | melanosome | 2.65E-03 |
| GO:0033631 | cell-cell adhesion mediated by integrin | 2.66E-03 |
| GO:0070527 | platelet aggregation | 2.99E-03 |
| GO:0008544 | epidermis development | 3.12E-03 |
| GO:0002183 | cytoplasmic translational initiation | 3.29E-03 |
| GO:0016282 | eukaryotic 43S preinitiation complex | 3.29E-03 |
| GO:0061077 | chaperone-mediated protein folding | 3.33E-03 |
| GO:0044183 | protein binding involved in protein folding | 3.50E-03 |
| GO:0001732 | formation of cytoplasmic translation initiation complex | 3.82E-03 |
| GO:0033290 | eukaryotic 48S preinitiation complex | 3.82E-03 |
| GO:0071438 | invadopodium membrane | 3.90E-03 |
| GO:0019960 | C-X3-C chemokine binding | 3.90E-03 |
| GO:0001533 | cornified envelope | 4.23E-03 |
| GO:0006412 | Translation | 4.23E-03 |
| GO:0005509 | calcium ion binding | 4.68E-03 |
| GO:0033627 | cell adhesion mediated by integrin | 5.20E-03 |
| GO:0015026 | coreceptor activity | 5.48E-03 |
| GO:0044267 | cellular protein metabolic process | 5.66E-03 |
| GO:0006458 | de novo protein folding | 6.00E-03 |
| GO:0010951 | negative regulation of endopeptidase activity | 1.00E-02 |
| GO:1901998 | toxin transport | 1.06E-02 |
| GO:0002009 | morphogenesis of an epithelium | 1.11E-02 |
| GO:0071711 | basement membrane organization | 1.12E-02 |
| GO:0005852 | eukaryotic translation initiation factor 3 complex | 1.24E-02 |
| GO:0031092 | platelet alpha granule membrane | 1.24E-02 |
| GO:0034667 | integrin alpha3-beta1 complex | 1.26E-02 |
| GO:0032212 | positive regulation of telomere maintenance via telomerase | 1.27E-02 |
| GO:0005581 | collagen trimer | 1.27E-02 |
| GO:0034774 | secretory granule lumen | 1.29E-02 |
| GO:0034113 | heterotypic cell-cell adhesion | 1.34E-02 |
| GO:0050731 | positive regulation of peptidyl-tyrosine phosphorylation | 1.35E-02 |
| GO:0048407 | platelet-derived growth factor binding | 1.35E-02 |
| GO:0005577 | fibrinogen complex | 1.35E-02 |
| GO:0042730 | Fibrinolysis | 1.47E-02 |
| GO:0030529 | ribonucleoprotein complex | 1.64E-02 |
| GO:0060135 | maternal process involved in female pregnancy | 1.64E-02 |
| GO:0004175 | endopeptidase activity | 1.80E-02 |
| GO:0031258 | lamellipodium membrane | 1.80E-02 |
| GO:0050750 | low-density lipoprotein particle receptor binding | 1.80E-02 |
| GO:0050821 | protein stabilization | 1.84E-02 |
| GO:0004812 | aminoacyl-tRNA ligase activity | 1.94E-02 |
| GO:0043588 | skin development | 2.06E-02 |
| GO:1904851 | positive regulation of establishment of protein localization to telomere | 2.06E-02 |
| GO:0033622 | integrin activation | 2.06E-02 |
| GO:0050839 | cell adhesion molecule binding | 2.08E-02 |
| GO:0001666 | response to hypoxia | 2.16E-02 |
| GO:0044877 | macromolecular complex binding | 2.30E-02 |
| GO:0070419 | nonhomologous end joining complex | 2.47E-02 |
| GO:0030280 | structural constituent of epidermis | 2.47E-02 |
| GO:0009897 | external side of plasma membrane | 2.74E-02 |
| GO:0006418 | tRNA aminoacylation for protein translation | 2.74E-02 |
| GO:0007159 | leukocyte cell-cell adhesion | 2.74E-02 |
| GO:0010710 | regulation of collagen catabolic process | 2.74E-02 |
| GO:0030023 | extracellular matrix constituent conferring elasticity | 2.74E-02 |
| GO:1990498 | mitotic spindle microtubule | 2.74E-02 |
| GO:0050900 | leukocyte migration | 2.84E-02 |
| GO:0051918 | negative regulation of fibrinolysis | 2.85E-02 |
| GO:0005044 | scavenger receptor activity | 3.34E-02 |
| GO:0016477 | cell migration | 3.36E-02 |
| GO:0031581 | hemidesmosome assembly | 3.36E-02 |
| GO:0016504 | peptidase activator activity | 3.36E-02 |
| GO:0004003 | ATP-dependent DNA helicase activity | 3.85E-02 |
| GO:0043394 | proteoglycan binding | 3.99E-02 |
| GO:0017025 | TBP-class protein binding | 4.15E-02 |
| GO:0004866 | endopeptidase inhibitor activity | 4.51E-02 |
| GO:0010952 | positive regulation of peptidase activity | 4.60E-02 |
| GO:0048333 | mesodermal cell differentiation | 4.60E-02 |
| GO:0006521 | regulation of cellular amino acid metabolic process | 4.71E-02 |
| GO:0043260 | laminin-11 complex | 4.71E-02 |
| GO:0005606 | laminin-1 complex | 4.71E-02 |
| GO:0007161 | calcium-independent cell-matrix adhesion | 4.71E-02 |
| GO:0043564 | Ku70:Ku80 complex | 4.71E-02 |
